# Effects of pH and metal composition on selective extraction of calcium from steel slag for Ca(OH)_2_ production

**DOI:** 10.1039/d0ra08497b

**Published:** 2021-02-22

**Authors:** Ye Hwan Lee, Hanki Eom, Sang Moon Lee, Sung Su Kim

**Affiliations:** Department of Environmental Energy Engineering, Graduate School of Kyonggi University 94-6 San, Iui-dong, Youngtong-ku Suwon-si Gyeonggi-do 442-760 Korea; Department of Environmental Energy Engineering, Kyonggi University 94-6 San, Iui-dong, Youngtong-ku Suwon-si Gyeonggi-do 442-760 Korea sskim@kyonggi.ac.kr

## Abstract

This research article explains the effects of pH and metal composition on the selective calcium extraction from steel slag. The operating parameters including extraction solvent type, solvent concentration, metal composition of steel slag, substance type and pH were investigated. HCl, NH_4_Cl, NH_4_OH and NaOH were employed as solvents to extract Ca from steel slag. It has been shown that hydrochloric acid effectively extracts Ca. The high metal content in steel slag reacted sensitively to the solvent concentration, and a specific concentration was derived to selectively extract Ca. The optimal solvent for calcium extraction was 2 M HCl, which induced the extraction of 97% of Ca; 46% of Mg; 35% of Al; and 1% of Si from the steel slag. In order to separate Ca in the leaching solution from other metal ions, various acidic/basic substances were added to regulate the pH. The optimal pH level for removing the impurities without calcium was found to be 9.5. The precipitated impurities were removed by filtration, and the pH was adjusted to 13 or higher for Ca(OH)_2_(s) production. In conclusion, scanning electron microscopy (SEM) and energy-dispersive X-ray spectroscopy (EDX) revealed that the Ca content produced through the process was more than 99%. It is expected that high-purity Precipitated Calcium Carbonate (PCC) will be achieved when the generated Ca(OH)_2_ is used as a source of calcium for mineral carbonation.

## Abstract 2

This research article explains the effects of pH and metal composition on the selective calcium extraction from steel slag.

## Graphical abstract

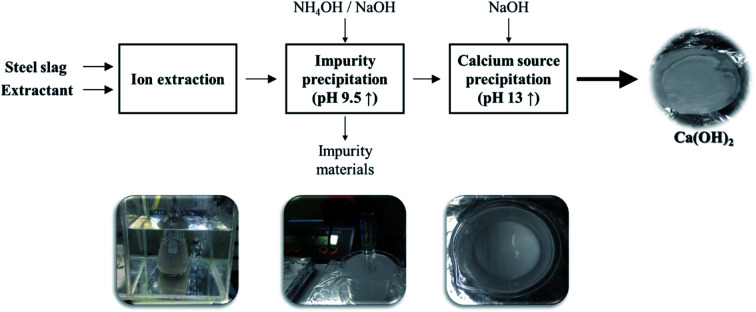

## 1. Introduction

In the 1750s, at the beginning of the Industrial Revolution, the CO_2_ concentration in the atmosphere was approximately 280 ppm (±10), 415 ppm was measured in the year of 2018.^1^ The elevation of CO_2_ levels has brought concerns about anthropogenic CO_2_ emissions, due to the increased energy demand in both developed and developing worlds.^2^ The long-term climate change is also believed to be strongly related to the increased atmospheric CO_2_ concentration due to anthropogenic emissions.^3^ In order to minimize the impact of CO_2_ on climate change, technologies to reduce CO_2_ in the atmosphere are being developed. The most popular technology employed is Carbon Capture & Storage (CCS), which absorbs, captures and stores the CO_2_ generated in the atmosphere.^4–7^ However, CCS problems such as transportation for storage, storage space, and stability have emerged. This is because the captured CO_2_ is transported from the emission source to the storage space (geological storage *etc.*) and may leak even after storage. Therefore, a technology capable of stably treating CO_2_ directly from an emission source is required.

The top five sectors in the industry where CO_2_ emissions are high: (1) the power industry; (2) cement production; (3) refineries; (4) the iron and steel industry; and (5) the petrochemical industry.^8^ Recently, iron and steel industry has tried to reduce CO_2_ by using mineral carbonation. Mineral carbonation can reduce CO_2_ and waste from iron and steel industry.

Mineral carbonation refers to the production of environmentally harmless carbonate where CO_2_ and calcium or magnesium are involved during the chemical reaction.^9,10^ Since the energy level of carbonates used in the carbonation process is about a 60–180 kJ mol^−1^ lower than that of CO_2_ (400 kJ mol^−1^), the mineral carbonation method is thermodynamically stabilized, leak-free, environmentally friendly CO_2_ treatment process.^11–15^ There is direct and indirect carbonation in the mineral carbonation process. During direct carbonation, CO_2_ is injected into minerals to produce carbonates while indirect carbonation involves the extraction of alkali ions or Ca and Mg in the minerals. The rate of direct carbonation reaction is slow and high pressure and temperature are required.^16,17^ On the other hand, indirect carbonation has two sequential processes: the extraction of alkali ions such as Ca or Mg in the minerals; and the production of carbonates. Although the process is more complicated than that of direct carbonation, indirect carbonation is more feasible in producing high-purity carbonates and can be easily extended to the level of commercialization.^12,18^

For the past few years, studies have focused on the use, not of the natural minerals, but the industrial by-products as the raw materials for mineral carbonation. Slag, a major waste in the steel industry, is produced at 130 million tons per year, and is mostly landfilled.^19,20^ But slag containing large quantity of calcium can be used as an appropriate material for mineral carbonation.^21,22^ Therefore, carbonation using industrial by-products is a reasonable technique to reduce waste and carbon dioxide. Carbonation using industrial by-products has been studied in various extraction methods to improve calcium extraction efficiency. This is because the higher extraction efficiency can capture more CO_2_. It also minimizes waste of resources. Table 1 summarizes the calcium extraction technology from recently reported slag.

**Table 1:** Mineral carbonation–extraction process using slag

Slag type	Particle size	Ca content	Reaction temp.	S/L ratio	Reaction time	Solvent	Ca extraction	Impurity ion extraction	Ref.
Steel slag	50–74 μm	CaO 44.9% (XRF)	Room temperature	20 g L^−1^	60 min	1 M NH_4_Cl	96%	—	23
Steelmaking slag	—	15.42% (EDS)	45 °C	100 g L^−1^	30 min	2 M NH_4_Cl	76%	Mg: 255 mg L^−1^	24
Steel converter slag	138.3 μm	CaO 41% (XRF)	80 °C	136.8 g L^−1^	60 min	0.5 M succinic acid	30%	Mg: 31%	25
Blast furnace slag	138.3 μm	CaO 41% (XRF)	30 °C	136.8 g L^−1^	60 min	2 M CH_3_COOH	90%	Al: 38%, Si: 7.6%	26
Steelmaking slag	≤250 μm	CaO 51.4% (XRF)	Room temperature	100 g L^−1^	30 min	1 M NH_4_Cl	78%	—	27
Blast furnace slag	50.43 μm	CaO 38.95% (XRF)	80 °C	1/4	60 min	(NH_4_)_2_SO_4_	100%	Mg: 100%, Al: 100%	28
Basic Oxygen Steelmaking (BOS) slag	≤150 μm	CaO 34.4% (XRF)	—	100 g L^−1^	120 min	2 M NH_4_Cl	8457.6 mg L^−1^	S: 197.1 mg L^−1^, Mg: 75.1 mg L^−1^, V: 5.5 mg L^−1^	18
Secondary Steelmaking (SS) slag	≤150 μm	CaO 38.2% (XRF)	—	100 g L^−1^	120 min	2 M NH_4_Cl	8369 mg L^−1^	S: 180.7 mg L^−1^, Mg: 57.6 mg L^−1^, V: 2.9 mg L^−1^	18
Hot Metal Desulfurisation (HMD) slag	≤150 μm	CaO 39.3% (XRF)	—	100 g L^−1^	120 min	2 M NH_4_Cl	11 189.9 mg L^−1^	S: 607.3 mg L^−1^, Mg: 57.6 mg L^−1^, V: 3.6 mg L^−1^	18

Typical extraction solvents used for the extraction of calcium are acidic substances such as acetic acid and hydrochloric acid. When acetic acid was used to extract calcium, long filtration processes were required due to the higher viscosity of the slurry than HCl extractant which will demand longer processing period and higher energy consumption. However, a large amount of impurities is created during the extraction process. Ammonium chloride can selectively extract calcium, but it should be used at a concentration of 1 M or less. However, the extraction efficiency of calcium is lowered by using a low concentration of the extraction solvent. Irfan *et al.*^29^ used 4.13 M of HNO_3_ for calcium leaching from cement kiln dust. However, the removal of the extracted impurities along with calcium was not discussed. The two most important factors in the extraction process for industrial practice which enables recycling of the calcium as a resource are calcium extraction efficiency and selectivity. Since those enable high purity carbonate production, and efficient CO_2_ capture. Therefore, a technique capable of removing impurities and extracting calcium is critical.

As shown in previous studies, various impurities like Mg, Al, Si, or their ions are extracted during the calcium extraction from the steel slag. These impurities consequently lower the purity of carbonates produced during the carbonation process and depreciate their economic value. Hence, a significant amount of efforts has been made to separate the impurities for improvement of carbonate purity.^3,11,30^ Crom *et al.*^3^ reported regulating temperature and pH for impurities (Mg, Al, Si) removal, which are extracted along with calcium during the extraction of calcium in a blast furnace slag. Wang *et al.*^30^ reported the removal of impurities (Fe, Al, Cr, Zn, Cu, Mn) by adding aqueous ammonia to obtain high purity magnesium carbonate. Azdarpour *et al.*^11^ injected ammonium hydroxide to regulate pH for precipitation of carbonates after extracting calcium from red gypsum. As a result, one of the major impurities, Fe, was successfully separated. Many studies have reported that impurities are removed through the regulation of pH. However, the optimized pH is different, and it is unknown whether it can be applied to the slag of different compositions. In addition, the reported study focused only on the removal of impurities and did not address the calcium phase Ca(OH)_2_ production for the carbonation reaction.

Recently, many studies on mineral carbonation technology using Ca(OH)_2_ have been reported.^31,32^ Kang *et al.*^31^ reported that during CO_2_ capture using adenosine monophosphate (AMP), mixing of Ca(OH)_2_ instead of CaCl_2_ showed superior performance in CO_2_ desorption and sorbent regeneration. The Ca(OH)_2_ employed in these studies is reagent grade. Therefore, it is necessary to achieve the recovery of high purity Ca(OH)_2_ from industrial by-products, which would be beneficial for the manufacture of carbonate.

Therefore, in this study has focused on phase of the calcium source for carbonation as well as removing impurities. Ca(OH)_2_ was obtained through a pH swing process for removing impurities. The characteristics of extraction by different extractants have been investigated. The pH for Ca extraction of was controlled by the acidic and alkali solution and the relationship between pH and precipitation of impurities were characterized. In addition, the pC–pH diagram was examined to demonstrate the dissolution characteristics of ion and the validity of the results. Based on this, the optimal pH for impurities and Ca separation in the form of Ca(OH)_2_ was derived.

## 2. Material and methods

### 2.1. Ion composition of the steel slag

Four types of steel slag with different compositions were employed, and the slag occurred from the steelmaking process in Korea. To confirm the ion composition in steel slag, inductively coupled plasma ICP (Waters 600E/431/125, Waters) analyses was used. The ions of metal elements such as Ca, Mg, Al, Si, and Fe were found from spectroscopy. The contents are shown in Table 2. The efficiency of extraction was estimated by comparing the result of ICP.

**Table 2:** Ion composition in various steel slag (ICP)

Elements	Steel slag			
	Slag 1 (S1)	Slag 2 (S2)	Slag 3 (S3)	Slag 4 (S4)
	ICP (%)	ICP (%)	ICP (%)	ICP (%)
Mg	6.50	2.61	3.23	4.85
Al	4.77	3.53	3.85	10.98
Si	7.24	4.82	3.83	10.28
Ca	30.98	11.90	10.13	30.60
Fe	—	39.86	30.61	—

### 2.2. Ion extraction and separation

To extract calcium from steel slag, 10 g of a slag particle size of 850 μm or smaller and 100 mL of extractant were injected into an Erlenmeyer-flask and vigorously stirred at room temperature for 10 min. To confirm extraction efficiency at different types of extractant, HCl (35–37%, Samchun Co.), NH_4_Cl (98.5%, Samchun Co.), NaOH (98%, Samchun Co.), and NH_4_OH (28%, Junsei Co.) were diluted in deionized water (D.W.) to various concentrations (0.7, 1.2, 1.5, 2 M) and used as extractants. To separate the solid from the slurry after the reaction, glass microfiber filters (GF/C filters, Whatman Co., pore size; 0.45 mm, diameter; 47 mm) was used. To confirm the amount of various extracted ions in the obtained extracts, ICP was conducted.

To separate various impurities (Mg, Al, Si) extracted from steel slag along with calcium, acidic/basic substances were injected into the extract. The precipitation generated by the reaction with the acidic/basic substances were separated by using a centrifuge and filtration. The properties of the precipitation acquired by the filtration were characterized by X-ray diffraction (XRD; X'Pert PRO MRD, PANalytical) analysis in the 2*θ* range of 10–90° at a scan rate of 6° min^−1^ and SEM/EDX mapping (S-4800, Hitachi).

### 2.3. Ion solubility at different pH levels

The solubility of cations (Ca, Mg, Al and Si) in the pH range of 0.5 to 13.5 were characterized. The cation was extracted from chlorine compounds using HCl, and the pH was regulated by NaOH. The upper part of the solution after filtration was taken for measuring the concentration.

## 3. Result and discussion

**Fig. 1:**
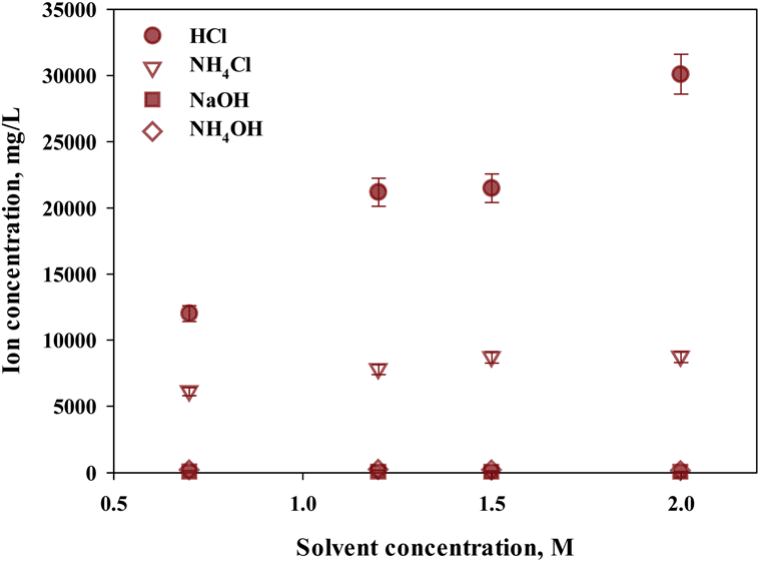
Dissolved Ca and other ions in various extractants and concentrations.

Ca extraction was carried out by 4 types of solvents (HCl, NH_4_Cl, NaOH, NH_4_OH). NH_4_Cl is commonly used Ca extractant. HCl was chosen to compare the performance since it is also well-known Ca extractant. NH_4_OH (weak base) and NaOH (strong base) were selected as controls to compare the pH effects of extracts during calcium extraction. Acetic acid was excluded because it was known to have a low filtration rate because of the high viscosity of the reactants. The concentration of the extraction solvent used in the experiment was 0.7–2 M, and the results are shown in Fig. 1. Hydrochloric acid showed the highest extraction performance among them, and the extraction quantity increased with increasing concentration. On the other hand, NH_4_Cl extracted relatively lower quantity of calcium, and the effect of concentration was minimal.

As expected, sodium hydroxide and ammonium hydroxide showed very low effectiveness in terms of extraction, regardless of the concentration. When using a 2 M extractant, the amounts of extraction were HCl (30 100 mg L^−1^, 97%); ammonium chloride (8725 mg L^−1^, 28%); sodium hydroxide (18 mg L^−1^, 0%); and ammonium hydroxide (106 mg L^−1^, 0%). The trends suggest the effect of anion binding calcium and the pH level. When using an extractant that contains chloride, the amount of Ca extracted was higher than a hydroxide containing extractant. When using a hydroxide extractant, almost no calcium extraction was observed. When chloride extractant was used, calcium chloride was generated during calcium extraction, of which solubility was 74.5 g/100 mL (20 °C). On the other hand, an extractant including hydroxide created calcium hydroxide during the extraction of calcium, of which solubility was very low at 0.173 g/100 mL (20 °C). Therefore, it is believed that the products from the reaction differ according to the different types of extractant. In addition, the pH of the extractant and solubility of products affect the efficiency of calcium extraction. Nevertheless, NH_4_Cl was used nevertheless for the regeneration of the extraction solvent. When the ammonium solution was attempted in the previous study, it showed the extractant can be reused.^27^ However, in order to maximize the use of slag with low efficiency, a huge reactor is required. This challenge can be solved by using HCl. Recently, in mineral carbonation process using CKD (Cement kiln dust), an electrochemical process to regenerate extraction solvents and alkaline substances has been attempted.^33^ This is because NaOH and HCl can be regenerated through alkali electrolysis processes.^34^ The NaCl solution can be used as an anolyte in the membrane electrolysis for NaOH production. Chlorine (Cl_2_) and hydrogen (H_2_) gases are acquired from anode and cathode, respectively, and they can be used to synthesize HCl.^33^ Therefore, in this study, HCl was selected as the optimal extractant to extract calcium from steel slag.

**Fig. 2:**
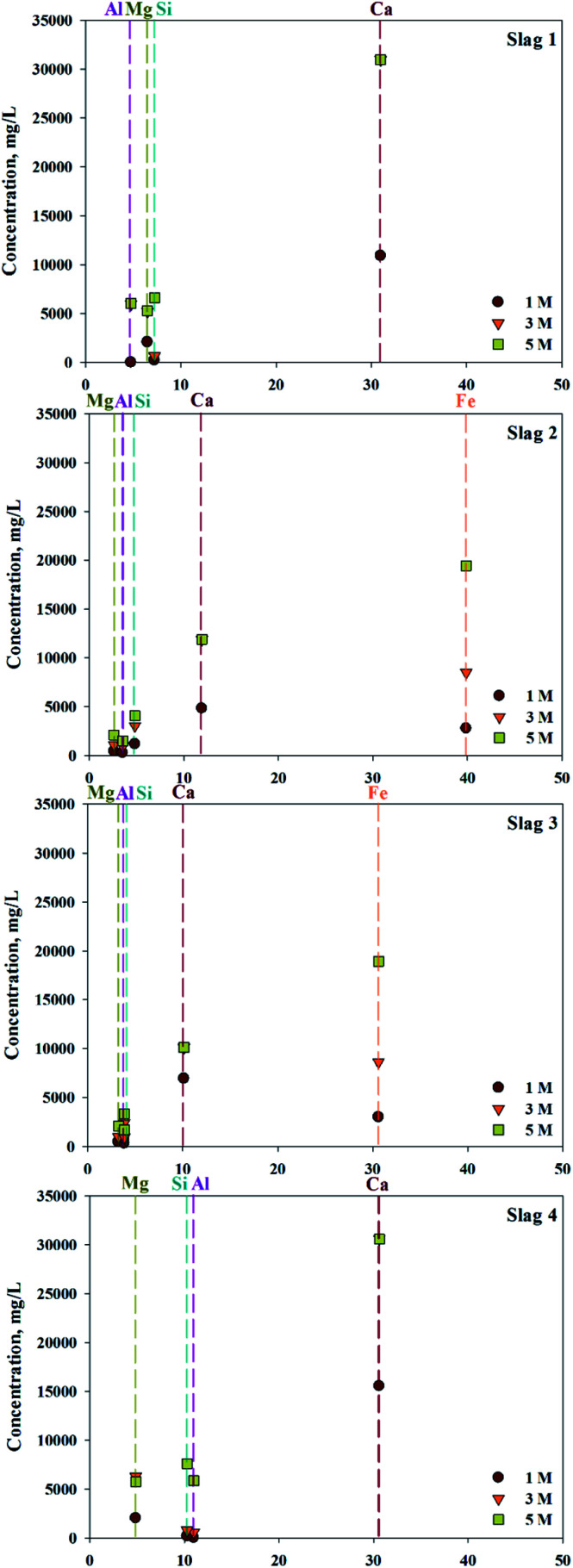
Relationship between ion composition of slag and extractant concentration.

ICP confirmed that various types of ions, in addition to calcium, exist in 4 types of slag (S1–4). To confirm the concentration of extractant and the extraction trends, different concentrations of HCl solutions were used as the extractant. The efficiency of extraction is shown in Fig. 2. Based on extraction trends observed at different concentrations, the amount of Ca extracted was 100% when above 3 M of HCl were used as an extractant. In cases of S1 and S4, where the content of Fe was low, there was little difference in the quantities of extracted impurities. On the other hand, in S2 and S3, where the Fe content was high, the amount of Fe extracted rapidly increased with increased extractant concentration. When 5 M HCl extractant was used, the amount of Fe extracted exceeded the amount of extracted Ca, indicating that the extractant used was not a suitable source of extraction. Overall, even the same concentration of extraction solvent is used, a small amount of calcium and a large amount of impurities are extracted if a source contains a large amount of impurities. Consequently, the composition of the source extraction and the concentration of extractant were important factors in the production of highly pure CaO or Ca(OH)_2_.

**Fig. 3:**
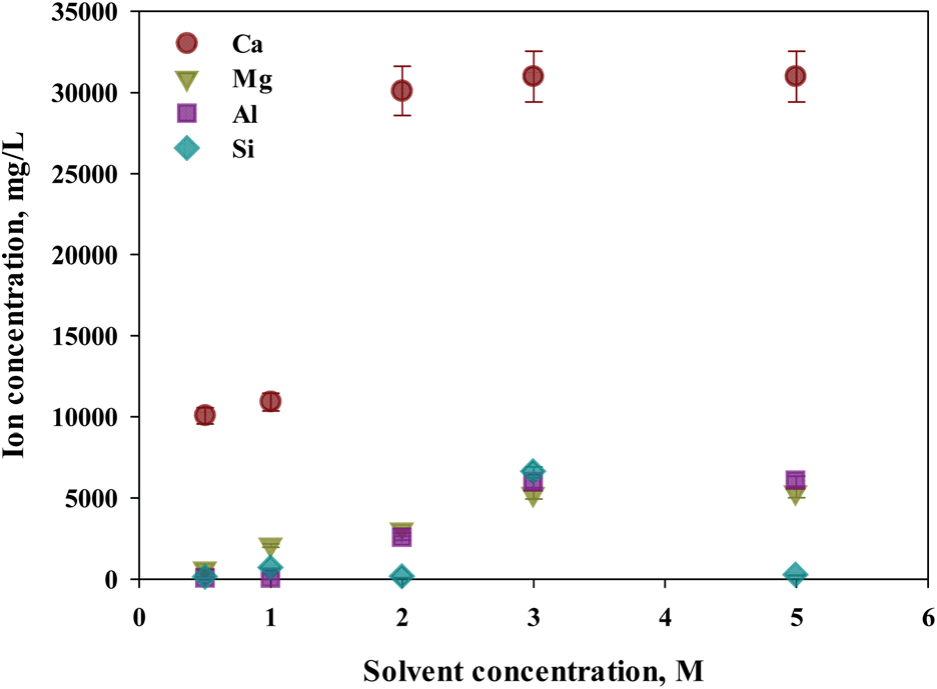
Dissolved Ca and other ions concentrations at various extractant concentrations.

Eloneva *et al.*^35^ reported that calcium could be selectively extracted according to the concentration of extractant during calcium-based extraction from steel slag. To determine an optimal concentration of extractant, increased concentrations of HCl (from 0.5 to 5 M) were used as extractants. S1 was used as the source of calcium extraction due to the highest calcium content. As shown in Fig. 3, the major components of steel slag, which are Ca, Mg, Al, and Si were all extracted with this process. The increased extraction was achieved with higher concentrations of extractant. The maximum extraction was found from calcium concentrations of 2 M or higher. In the case of impurities, the efficiency of extraction was approximately 80% at 3 M or higher HCl solutions. Hence, 2 M HCl was believed to be the optimal extractant to promote the selectivity of calcium.

**Fig. 4:**
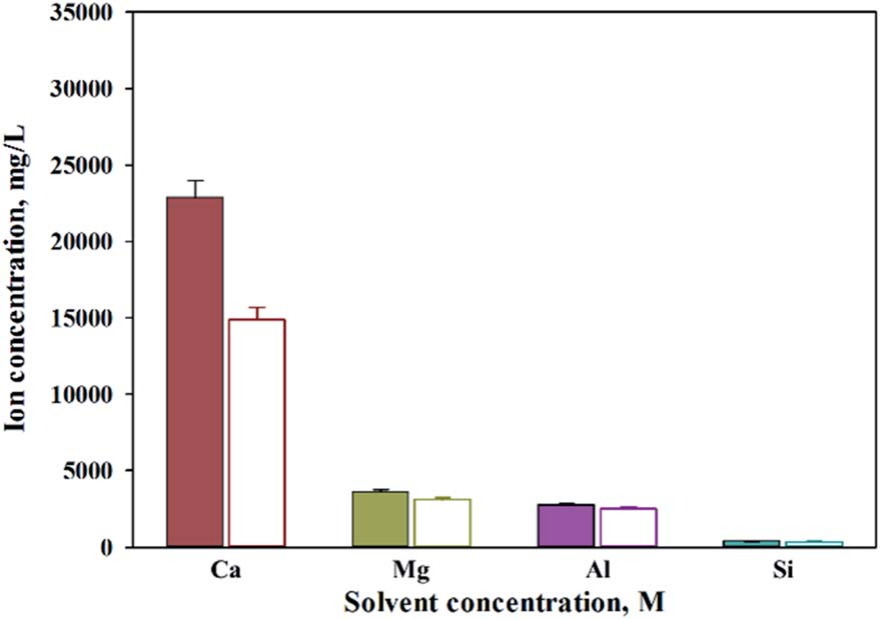
Dissolved Ca and other ions concentrations when injected with sulfuric acid (filled: extract, opened: injected H_2_SO_4_).

**Fig. 5:**
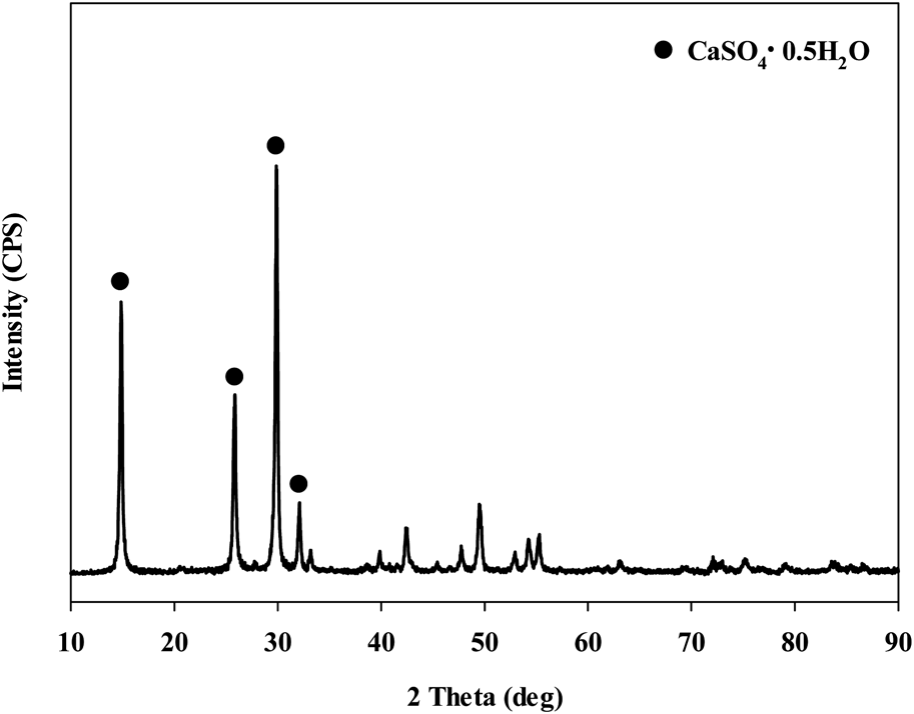
XRD patterns of the samples after injected with sulfuric acid.

When extracting calcium in steel slag using an extractant, the pH swing method was used to separate impurities which were extracted along with calcium. To regulate the pH level of extract, acidic/basic substances were injected. Sulfuric acid and nitric acid were the acids injected, while sodium hydroxide and ammonium hydroxide were used as the bases. The pH swing experiment was conducted using acid after the extraction of ions in steel slag, with the use of a 2 M HCl solution as the extractant. Precipitation was generated when sulfuric acid was injected while no precipitation occurred when nitric acid was injected. Fig. 4 shows the result of pH swing using sulfuric acid. Fig. 5 shows the XRD pattern of the generated substances. The reductions found when injecting sulfuric acid were approximately: 34.8% (7950 mg L^−1^) for Ca; 14% (500 mg L^−1^) for Mg; 7% (195 mg L^−1^) for Al; and 0.13% (0.5 mg L^−1^) for Si. The XRD results showed that Ca was precipitated as the form of CaSO_4_. Since the purpose of the study is to produce highly pure CaO or Ca(OH)_2_ through the removal of impurities, the injection of sulfuric acid (H_2_SO_4_) was deemed unsuitable due to the formation of CaSO_4_ although selective separation by precipitation was possible.

In addition, methods for removing impurities have been reported. The regulation of pH at 8.5 to 8.6 by using NH_4_OH led to the removal of impurities, including Fe.^30,36,37^ Hemmati *et al.*^38,39^ used HCl to remove impurities by using NaOH after the extraction of Mg in serpentine, and Hu *et al.*^28^ used (NH_4_)_2_SO_4_ to regulate pH at 5.5 after the extraction of Mg and Ca, capable of separating 99% of Al (with 2% co-precipitation of Mg) by precipitation. Crom *et al.*^3^ separated Si by precipitation *via* lowering the temperature from 20 to 1 °C and selectively separated Al (92%) and Mg (64%) by controlling the pH from 4.4 to 8.4.

**Fig. 6:**
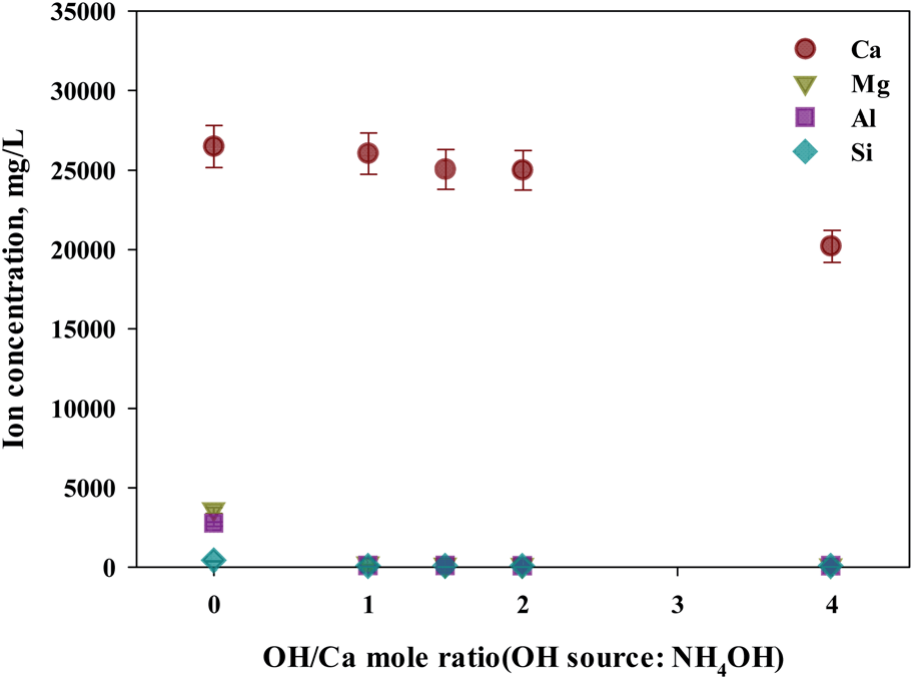
Dissolved Ca and other ions concentrations at various injection quantities of NH_4_OH.

**Fig. 7:**
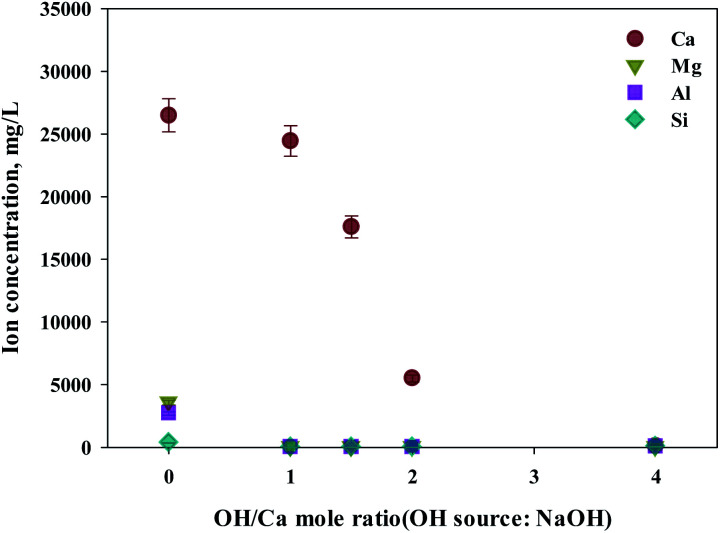
Dissolved Ca and other ions concentrations at various injected quantities of NaOH.

As shown in this study, precipitation was generated when basic substances were injected. The color of precipitation changed depending on the amount of substance injected. For the fine control of pH, a weak (NH_4_OH) and, a strong base (NaOH) was used. NaOH and NH_4_OH were injected in accordance with the ratio of Ca^2+^ : OH^−^ to confirm the separation of calcium and impurities by precipitation as shown in Fig. 6 and 7. Most of the other ions precipitated when injecting NH_4_OH, with decreased calcium by up to 23.7% (6300 mg L^−1^, 1 : 4 ratio).

Meanwhile, when NaOH was injected, most of the other ions precipitated. However, calcium was also found in the precipitation. Further investigation was conducted to understand the occurrence of calcium in the precipitation, the pH resulting from the different basic substances was explored. In the injection of NH_4_OH, pH was approximately 10.5 (1 : 4 ratio). When NaOH was injected, pH was approximately 13 (1 : 4 ratio). In the case of calcium, most ions precipitated at pH 13 while other ions precipitated at pH 9.6.

**Fig. 8:**
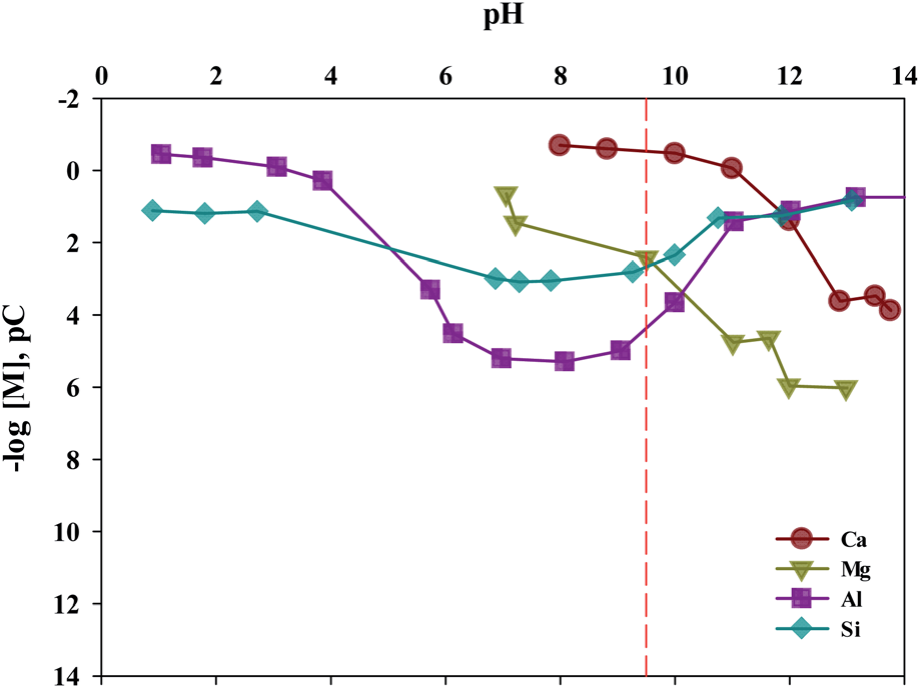
Equilibrium pC–pH diagram for Ca, Mg, Al and Si.

The composition of industrial waste being used for mineral carbonation is all different by samples, and the concentration of impurities varies depending on the extraction conditions. Therefore, the optimum pH for removing impurities is different. In this study, the dissolution properties for substances were investigated by a different level of pH. Moreover, in this study, fine tuning of pH levels was performed to verify the solubility of ion, as the process parameters for separating the various ions are controlled by using different pH levels. The result is shown in Fig. 8. It is to be noted that the *K*_sp_ of Ca(OH)_2_, Fe(OH)_2_, Fe(OH)_3_, Al(OH)_3_ and Mg(OH)_2_ are about 6.5 × 10^−6^, 8.0 × 10^−16^, 2.79 × 10^−39^, 3 × 10^−34^ and 5.61 × 10^−12^, respectively. Thus the Ca(OH)_2_ is confirmed to dissolve much more readily than that of other ions. In addition, Fe(OH)_2_, Fe(OH)_3_, Al(OH)_3_ and Mg(OH)_2_ were found to precipitate faster than Ca(OH)_2_ at alkaline pH. In cases of Ca and Mg, while their solubilities decreased as the pH increased, the solubilities of Si and Al increased when the pH level was either at the range of a strong acid or a strong base. Based on the results acquired, the solubilities according to pH (between 9 and 10) were calculated. The optimal pH for the separation of impurities was found to be 9.5.

**Fig. 9:**
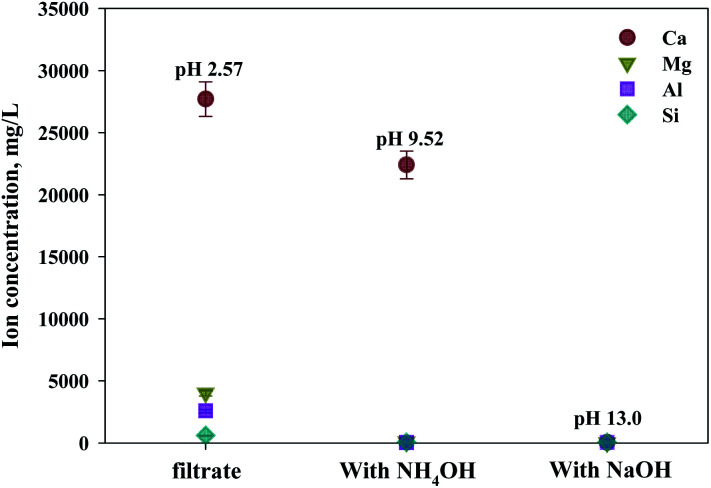
Dissolved Ca and other ions concentrations at various injected quantities of basis materials.

**Fig. 10:**
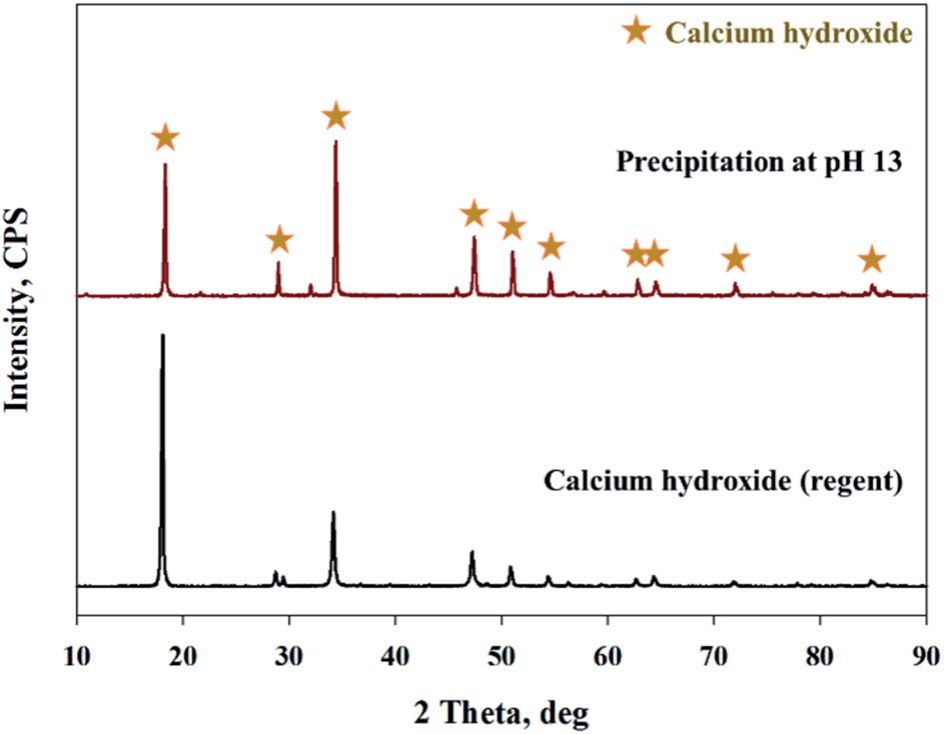
XRD patterns of precipitation at pH 13 and calcium hydroxide (Ca(OH)_2_).

**Fig. 11:**
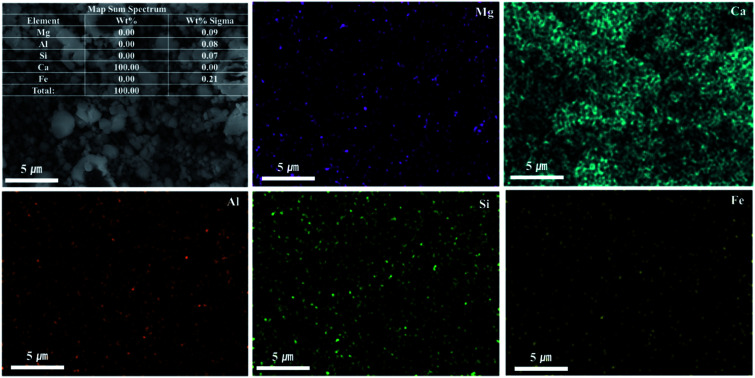
SEM/EDX mapping of precipitation at pH 13.

As shown in Fig. 9, Ca(OH)_2_ recovery experiments were carried out under optimal conditions. The pH of the filtrate was 2.57 after extraction of the slag with hydrochloric acid at 2 M with pH adjusted to 9.5 with NH_4_OH. Most impurities were removed, and the calcium concentration on the solution was 22 400 mg L^−1^. After filtration of impurities, NaOH was injected to increase the pH to 13 to obtain a solid calcium compound. The yield of calcium compounds recovered from the slag was 72% and yield showed 99.9% from leachate after removing impurities. To identify the precipitation generated at pH 13, XRD was conducted. As shown in Fig. 10, it was found that Ca precipitated in the form of Ca(OH)_2_. This indicates that separation by precipitation is feasible by using the regulation of pH. Fig. 11 shows the results of SEM/EDX analysis conducted to identify surface properties of precipitation at pH 13. Precipitation contained almost spherical particles sizes ranging 0.5–5 μm. EDX/EDX mapping shows that Ca ions were mostly observed on precipitation surface. The composition of precipitation was over 99% of Ca and about 1% of various metal ions (Mg, Al, Si, Fe). The generated Ca(OH)_2_ is expected to be able to provide a source of calcium extraction in the mineral carbonation process as well as production of high-purity PCC. The cost for recovering Ca(OH)_2_ from slag could be reduced by waste treatment and greenhouse gas reduction cost.

## 4. Conclusions

According to the results, 2 M HCl was found to be an excellent extractant; the efficiency for cation extraction from steel slag is the highest and it is capable of selectively extract calcium. When using 2 M HCl as the extractant, 97% of Ca was extracted, along with Al (35%), Mg (46%), and Si (1%) as impurities.

In effort to enhance the selectivity of Ca by removing the impurities, acidic/basic substances were injected. At pH 9.6, most of the impurities (Mg, Al, Si), excluding Ca, were precipitated. To optimize the conditions, experiments on solubility in accordance with different pH levels were conducted. The result showed that Ca showed the highest purity at a pH of 9.5.

To produce high-purity Ca as part of Ca(OH)_2_, the pH was regulated to 13. Here, it was found that most Ca was transformed into Ca(OH)_2_. The yield of calcium compound recovered from the slag or impurities removed from leachate is 72% and 99.9%. It is expected that the synthesized Ca(OH)_2_ can be provided as the source of calcium extraction for the production of high-purity PCC.

## Conflicts of interest

There are no conflicts to declare.

## Supplementary Material

## References

[cit1] Hashimoto K. , in Global Carbon Dioxide Recycling, SpringerBriefs in Energy, 2019, pp. 5–17

[cit2] Arce G. L. A. F., Carvalho J. A., Nascimento L. F. C. (2014). Ecol. Modell..

[cit3] De Crom K., Chiang Y. W., Van Gerven T., Santos R. M. (2015). Chem. Eng. Res. Des..

[cit4] Gibbins J., Chalmers H. (2008). Energy Policy.

[cit5] Choi S., Drese J. H., Jones C. W. (2009). ChemSusChem.

[cit6] Dooley J. J., Dahowski R. T., Davidson C. L. (2009). Int. J. Greenhouse Gas Control.

[cit7] Olajire A. A. (2010). Energy.

[cit8] Metz L. M. B. , Davidson O., Coninck H. D. and Loos M., Intergovernmental panel on climate change. Special report on carbon dioxide capture and storage, Cambridge University Press, Cambridge, 2005

[cit9] Bobicki E. R., Liu Q., Xu Z., Zeng H. (2012). Prog. Energy Combust. Sci..

[cit10] Seifritz W. (1990). Nature.

[cit11] Azdarpour A., Asadullah M., Mohammadian E., Junin R., Hamidi H., Manan M., Daud A. R. M. (2015). Chem. Eng. J..

[cit12] Sun Y., Yao M. S., Zhang J. P., Yang G. (2011). Chem. Eng. J..

[cit13] Eloneva S., Teir S., Salminen J., Fogelholm C. J., Zevenhoven R. (2008). Energy.

[cit14] Workshop MC, ed. R. B. Schmidt, Pittsburgh, USA, 2001

[cit15] Newall P. , Clarke P. S., Haywood S. J., Scholes H. M., Clarke H. and King N. R., Report PH2/17 for IEA Greenhouse Gas R&D Programme, 2000

[cit16] Assima G. P., Larachi F., Molson J., Beaudoin G. (2014). Chem. Eng. J..

[cit17] Daval D., Hellmann R., Martinez I., Gangloff S., Guyot F. (2013). Chem. Geol..

[cit18] Hall C., Large D. J., Adderley B., West H. M. (2014). Miner. Eng..

[cit19] van Zomeren A., van der Laan S. R., Kobesen H. B. A., Huijgen W. J. J., Comans R. N. J. (2011). Waste Manag..

[cit20] Humbert P. S., Castro-Gomes J. (2019). J. Cleaner Prod..

[cit21] Eloneva S., Said A., Fogelholm C. J., Zevenhoven R. (2012). Appl. Energy.

[cit22] Kim Y., Worrell E. (2002). Energy Policy.

[cit23] Said A., Mattila O., Eloneva S., Järvinen M. (2015). Chem. Eng. Process..

[cit24] Lee S. M., Lee S. H., Jeong S. K., Youn M. H., Nguyen D. D., Chang S. W., Kim S. S. (2017). J. Ind. Eng. Chem..

[cit25] Santos R. M., Chiang Y. W., Elsen J., Van Gerven T. (2014). Hydrometallurgy.

[cit26] Chiang Y. W., Santos R. M., Elsen J., Meesschaert B., Martens J. A., Van Gerven T. (2014). Chem. Eng. J..

[cit27] Said A., Laukkanen T., Järvinen M. (2016). Appl. Energy.

[cit28] Hu J., Liu W., Wang L., Liu Q., Chen F., Yue H., Liang B., Lü L., Wang Y., Zhang G., Li C. (2017). J. Energy Chem..

[cit29] Irfan M. F., Hossain S. M. Z., Tariq I., Khan N. A., Tawfeeqi A., Goeva A., Wael M. (2020). Mining, Metall. Explor..

[cit30] Wang X., Maroto-Valer M. M. (2011). ChemSusChem.

[cit31] Kang J. M., Murnandari A., Youn M. H., Lee W., Park K. T., Kim Y. E., Kim H. J., Kang S. P., Lee J. H., Jeong S. K. (2018). Chem. Eng. J..

[cit32] Vance K., Falzone G., Pignatelli I., Bauchy M., Balonis M., Sant G. (2015). Ind. Eng. Chem. Res..

[cit33] Youn M. H., Park K. T., Lee Y. H., Kang S. P., Lee S. M., Kim S. S., Kim Y. E., Ko Y. N., Jeong S. K., Lee W. (2019). J. CO_2_ Util..

[cit34] Teir S., Eloneva S., Fogelholm C. J., Zevenhoven R. (2009). Appl. Energy.

[cit35] Eloneva S., Teir S., Revitzer H., Salminen J., Said A., Fogelholm C., Zevenhoven R. (2009). Steel Res. Int..

[cit36] Park A.-H. A., Fan L.-S. (2004). Chem. Eng. Sci..

[cit37] Sanna A., Dri M., Maroto-Valer M. (2013). Fuel.

[cit38] Hemmati A., Shayegan J., Sharratt P., Yeo T. Y., Bu J. (2014). Chem. Eng. J..

[cit39] Hemmati A., Shayegan J., Bu J., Yeo T. Y., Sharratt P. (2014). Int. J. Miner. Process..

